# Intracellular Peptides in Cell Biology and Pharmacology

**DOI:** 10.3390/biom9040150

**Published:** 2019-04-16

**Authors:** Christiane B. de Araujo, Andrea S. Heimann, Ricardo A. Remer, Lilian C. Russo, Alison Colquhoun, Fábio L. Forti, Emer S. Ferro

**Affiliations:** 1 Special Laboratory of Cell Cycle, Center of Toxins, Immune Response and Cell Signaling—CeTICS, Butantan Institute, São Paulo SP 05503-900, Brazil; chris.biologia@gmail.com; 2 Proteimax Biotecnologia LTDA, São Paulo SP 05581-001, Brazil; andrea@proteimax.com; 3 Remer Consultores Ltda., São Paulo SP 01411-001, Brazil; remer@remer.com.br; 4 Department of Biochemistry, Chemistry Institute, University of São Paulo 1111, São Paulo 05508-000, Brazil; lilianrusso@gmail.com (L.C.R.); flforti@iq.usp.br (F.L.F.); 5 Department of Cell and Developmental Biology, University of São Paulo (USP), São Paulo 05508-000, Brazil; alison@usp.br; 6 Department of Pharmacology, Biomedical Sciences Institute, University of São Paulo (USP), São Paulo 05508-000, Brazil

**Keywords:** intracellular peptides, proteasome, epilepsy, endocannabinoid, cancer, drug discovery, obesity, insulin resistance

## Abstract

Intracellular peptides are produced by proteasomes following degradation of nuclear, cytosolic, and mitochondrial proteins, and can be further processed by additional peptidases generating a larger pool of peptides within cells. Thousands of intracellular peptides have been sequenced in plants, yeast, zebrafish, rodents, and in human cells and tissues. Relative levels of intracellular peptides undergo changes in human diseases and also when cells are stimulated, corroborating their biological function. However, only a few intracellular peptides have been pharmacologically characterized and their biological significance and mechanism of action remains elusive. Here, some historical and general aspects on intracellular peptides’ biology and pharmacology are presented. Hemopressin and Pep19 are examples of intracellular peptides pharmacologically characterized as inverse agonists to cannabinoid type 1 G-protein coupled receptors (CB1R), and hemopressin fragment NFKF is shown herein to attenuate the symptoms of pilocarpine-induced epileptic seizures. Intracellular peptides EL28 (derived from proteasome 26S protease regulatory subunit 4; Rpt2), PepH (derived from Histone H2B type 1-H), and Pep5 (derived from G1/S-specific cyclin D2) are examples of peptides that function intracellularly. Intracellular peptides are suggested as biological functional molecules, and are also promising prototypes for new drug development.

## 1. Introduction

Peptides are produced by cells from proteins synthesized specifically for this purpose, or as byproducts of protein metabolism. Many of the products formed in the first case are known cellular modulating agents that contribute to the maintenance of homeostasis of living organisms. These latter peptides are frequently designated neuropeptides or hormonal peptides such as insulin, vasopressin, opioid peptides, SAAS peptides, corticotrophin, neurotensin, among many others [1]. In the second case, in compartments other than those specialized in protein degradation, specific cellular mechanisms such as protein ubiquitination induce proteins to limited digestion generating intermediate peptides [2]. Cells can also produce peptides by directly translating small mRNA sequences [3,4]. However, most of the endogenous bioactive peptides identified and investigated to date come from proteolytic processing or degradation of proteins, compared to peptides generated directly from small mRNAs translation. Antigenic peptides are another well-known class of peptides that escape further lysosomal and extra-lysosomal proteolytic degradation, and are presented at the cell surface associated to either major histocompatibility complex (MHC) class I (MHC-I) or class II (MHC-II). It is quite fascinating that every self-protein is represented at the cell surface by a single peptide of 9–12 amino acids bound to MHC-I [5,6,7,8]. Therefore, cells have multiple pathways to generate functional peptides and mechanisms to select specific peptides to escape complete degradation.

## 2. Intracellular Peptides—A Brief Historical Retrospective

To the best of our knowledge, the first descriptions of intracellular peptides appeared in the scientific literature in 1957 and 1958, and at that time they were described in Gram-negative bacteria *Pseudomonas hydrophila* and yeast *Torula utilis*, without any findings on their biological or pharmacological function [9,10]. Two decades later, one functional intracellular peptide named “diazepam binding inhibitor” (DBI; TVGDVNTDRPGLLDL) derived from acyl-CoA-binding protein was identified, and its biological activity as a GABA receptor agonist was characterized [11,12]. Later, a yeast *Saccharomyces cerevisiae* intracellular peptide termed “a-Factor” was described and characterized as a mating pheromone [13]. This a-Factor mating pheromone is produced within the cytoplasm by a series of steps involving lipid attachment (prenylation), N-terminal proteolytic cleavages by Ste24p and Axl1p, and transport of the cytosol into the extracellular space by Ste6p [13]. After secretion by an unconventional secretory pathway, a-Factor binds to a specific receptor (Ste3p) and stimulates mating.

Lately, our group used site-directed mutagenesis to produce catalytically inactive forms of oligopeptidases thimet-oligopeptidase (EC 3.4.24.15; THOP1) and neurolysin (EC 3.4.24.16; Nln), which were used in a substrate-capture assay, aiming to identify natural substrates for these oligopeptidases [14,15]. These original assays identified a previously unknown group of 13 intracellular peptides [14]. The first intracellular peptide characterized to have pharmacological activity was hemopressin (PVNFKFLSH), which is a highly conserved peptide sequence derived from hemoglobin alpha-chain [16]. Further use of substrate-capture assay allowed the isolation of eight additional and novel intracellular peptides from mouse adipose tissue, which were shown to contain putative post-translational modification sites [17]. Two of these intracellular peptides (LVVYPWTQRY and VVYPWTQRY) containing a putative protein kinase C (PKC) phosphorylation site competitively inhibited the phosphorylation of a standard PKC substrate, and were suggested to participate in the metabolic changes observed in angiotensin-converting enzyme transgenic mice [17]. Furthermore, phosphorylation of peptides that were degraded by oligopeptidases THOP1 and Nln led to reduced degradation, whereas phosphorylation of peptides that interacted as competitive inhibitors of these enzymes altered only the K(i)’s [18]. Taken together, these data suggested for the first time that extra-lysosomal proteolysis by proteasomes and oligopeptidases could produce novel functional peptides, which may modulate protein interactions within cells [19]. Further studies using electron spray mass spectrometry and peptidomics techniques corroborated these initial findings, and thousands of novel intracellular peptides have now been identified in plants [20,21], yeast [22], zebrafish [23], rodents [24,25,26], human cell lines [27,28,29], and human tissues [30,31]. It is worth to mention that MHC proteins and immunoproteasomes emerge later in evolution than regular proteasomes [32]. Therefore, intracellular peptides appear in evolution earlier than MHC-I antigens, and their evolutionary presence among species, from plants to humans, corroborates their biological significance. On the other hand, pharmacological activities have been shown for several intracellular peptides (Table 1).

## 3. Intracellular Peptides Generation

One of the crucial questions about intracellular peptides refers to the enzymes involved in their synthesis and degradation. The major extra-lysosomal proteolytic system ubiquitously distributed among living organisms is the ubiquitin–proteasome system (UPS). Therefore, UPS was suggested to be responsible at least for the initial generation of intracellular peptides [19]. Indeed, the use of epoxomicin (an irreversible proteasome inhibitor) in human embryonic kidney 293T cells (HEK293T) caused a marked decrease in the levels of the vast majority of intracellular peptides [74]. On the other hand, treatment with bortezomib, a reversible proteasome inhibitor used in medical practice, causes increased levels of intracellular peptides in HEK293T and SH-SY5Y cells of human neuroblastoma [80]. A reduction in intracellular peptide levels was observed in a cell model of Huntington’s disease, which resembles that found after proteasome inhibition [81]. Further studies have tested a wider variety of irreversible and competitive proteasome inhibitors including carfilzomib, MG132, MG262, MLN2238, AM114, and clasto-lactacystin-β-lactone [29]. Only MG262 caused a substantial increase in intracellular peptide levels comparable with the effects of bortezomib, although carfilzomib and MLN2238 raised the levels of some intracellular peptides [29]. Acting subsequent to the proteasome, other intracellular peptidases may also be related to the metabolism of intracellular peptides. Potent inhibitors of tripeptidyl-peptidase 2 (butabindide) and cellular aminopeptidases (bestatin) did not substantially alter intracellular peptide levels [29]. A recent review article points to the fact that proteasomes in the organism are diverse, and that structurally different proteasomes are present not only in different types of cells, but also in a single cell [82]. Therefore, it is possible that inhibition of different proteasome pools can cause different effects on generation and degradation of intracellular peptides. However, it seems evident that proteolytic processing by proteasomes is essential for the regulation of intracellular peptide homeostasis.

Oligopeptidases THOP1 and Nln were suggested to contribute to increase the diversity of intracellular peptides. Overexpression of THOP1 in HEK293 cells decreased 5 intracellular peptides by more than 70% (mean proportions less than 0.3-fold of controls) and another 12 peptides decreased from 30 to 70% (mean ratios of 0.3–0.69); these peptides represent possible substrates of THOP1 *in vivo* [27]. In these same conditions, three peptides were increased (mean ratios higher than 1.3) by overexpression of THOP1, representing possible products *in vivo* [27]. The concentration of additional peptides remained unchanged, suggesting that these peptides were neither substrates nor products of THOP1 [27]. Further studies, using the knockdown of THOP1 by siRNA, confirmed the participation of this enzyme in the metabolism of intracellular peptides in HEK293T cells [83]. In these latter analyses, approximately 100 peptides were identified, with 20 peptides increasing in the experimental samples (ratio ≥ 1.80) representing possible natural substrates of THOP1. Six peptides decreased in the experimental samples and may represent natural products of THOP1. Eighty-seven peptides were considered unaltered and thus are neither substrates nor products of THOP1 [83]. Therefore, THOP1 acting downstream of the proteasome is capable of both generating and degrading specific intracellular peptides [27,83].

Participation of Nln in the metabolism of intracellular peptides was demonstrated in an animal model of C57BL6 knockout mice for Nln (KO; Nln^−/−^) [84,85]. Semi-quantitative peptidomics in Nln^−/−^ brain tissue found that of the 18 identified peptides showing slight changes, 5 are present in secretory vesicles suggesting that Nln can also metabolize neuropeptides. Three of the identified peptides were found to be fragments of mitochondrial proteins, and also corroborate previous suggestions that Nln is relevant for mitochondrial metabolism of peptides. Intracellular peptides from α- and β-hemoglobin are increased in Nln^−/−^ mouse brain, and decreased after *in vitro* incubation with recombinant Nln. *In vitro* analysis showed that RVD-hemopressin (also known as pepcan12) [37] and VD-hemopressin (longer hemopressin forms) are less hydrolyzed by Nln than RVDPVNFKLL and VDPVNFKLL (shorter hemopressin forms). RVD-hemopressin are increased in Nln^−/−^ when compared with the C57BL6 wild-type mice, whereas VD-hemopressin shows no alteration in Nln^−/−^ mice compared with wild-type mice [84,85]. These data suggest that Nln plays a biological role metabolizing hemopressin-containing peptides. In non-neuronal tissues such as skeletal muscle (gastrocnemius), two intracellular peptides derived from troponin I increased more than twofold, whereas in adipose tissue two different peptides derived from acyl-CoA-binding protein and hemoglobin alpha subunit increased more than twofold. In the liver, several endocannabinoid peptides containing the mouse hemopressin sequence decreased more than twofold. In the soleus muscle, only one peptide derived from the hemoglobin alpha subunit decreased more than twofold. Together, these data suggest that Nln metabolizes specific intracellular peptides in both neuronal and non-neuronal tissues [84,85].

The majority of intracellular peptides identified in mouse brain slices remain within cells [54]. However, approximately 15% (50 out of 344 identified) of the intracellular peptides identified in mouse brain slices can be secreted, including VD-hemopressin and RVDPVNFKL. However, at least 28 intracellular peptides identified were only found in the secreted media of cultured mouse brain slices, including hemopressin-derived peptides RVDPVNFKLL and RVDPVNF [54]. These data suggest that extracellular processing of secreted intracellular peptides can increase the number and complexity of intracellular peptides functions; the secretory mechanism and enzymes involved in the extracellular processing of intracellular peptides remains unknown [80]. Inhibition of a mammalian nervous-system-specific membrane proteasome complex with a cell-impermeable proteasome inhibitor has been shown to block the production of extracellular peptides and to attenuate neuronal-activity-induced calcium signaling [86]. Thus, it is possible that nervous-system-specific membrane proteasome complexes could be involved in the secretion of intracellular peptides.

Taken together, proteasomes and the oligopeptidases THOP1 and Nln were proposed to contribute to intracellular peptide proteolytic processing. Further studies are necessary to investigate additional peptidases that may contribute to intracellular peptide processing inside and outside the cells.

## 4. Intracellular Peptides Acting on G-protein Coupled Receptors

Hemopressin (PVNFKFLSH), the first intracellular peptide identified using the substrate-capture assay, is an inverse agonist of CB1R with antinociceptive action and also the ability to regulate food intake in animal models [14,33,34,35,80]. Gomes et al. [35,36] identified amino acid extension variants at the N-terminal of hemopressin in the brains of mice, which were defined as RVD- and VD-hemopressins. It is noteworthy that hemopressin-containing peptides (i.e., RVD- and VD-hemopressins) were the first peptides shown to have CB1R agonist endocannabinoid activities [35]. Subsequent work by several groups has shown that endogenous hemopressins are preferably RVD- and VD-hemopressins, although more than 20 peptides containing the hemopressin sequence have been identified in mouse brain extracts, and at least RVD-hemopressin was suggested to behave as negative allosteric modulator of CB1R [35,37]. Recently, Hofer et al. [38] have demonstrated the predominant presence of hemopressins in the adrenal medulla, as well as in catecholaminergic neurons of the rodent central nervous system, further suggesting their biological and physiological significance as endocannabinoids. Additional investigations have corroborated the analgesic [87,88,89] and anorexigenics [90,91] properties of hemopressins. Xapelli et al. [39] showed that hemopressin acting as a modulator of CB1R receptors enhances the differentiation of oligodendroglia in neural cultures containing stem cells derived from the subventricular region of newborn mice. These results further suggested that hemopressins have a physiological function as endocannabinoids, and could be used therapeutically to treat demyelinating diseases [39].

**Figure 1:**
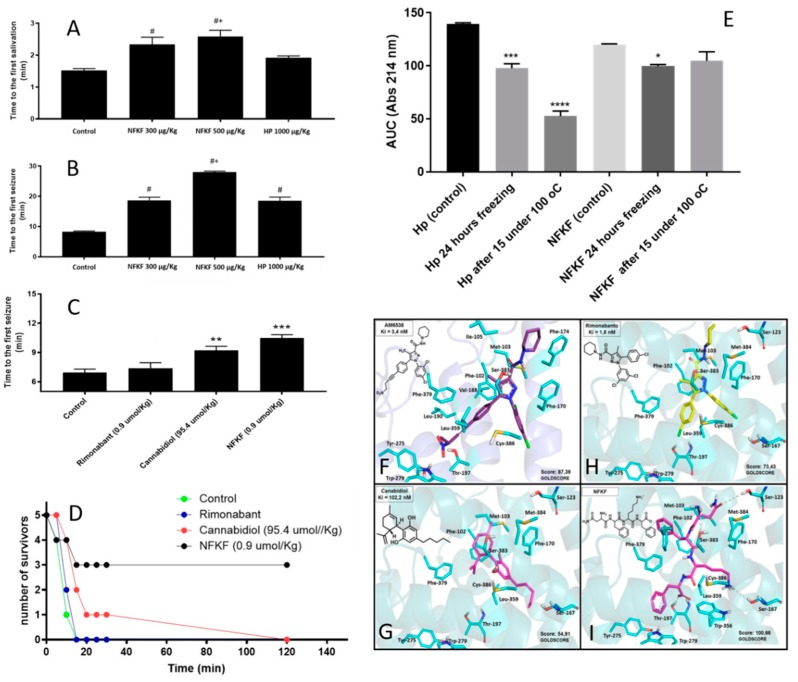
Pharmacological and molecular characterization of cannabinoids in cannabinoid type 1 G-protein coupled receptors (CB1R)-associated epilepsy treatment. (**A**–**D**) adult male C57BL/6J wild-type mice weighting approximately 24 g were kept in animal room with a controlled temperature (22 ± 1 °C) and a light–dark cycle of 12 h, and water and feed was supplied ad libitum. The number of animals used was the minimum necessary to obtain statistically significant results and they were maintained and used in accordance to the guidelines of the National Council for Control of Animal Experiments (CONCEA), following international norms of animal care and maintenance (Ethics protocol number ICB/USP 5100050218). Induction of epilepsy was by intraperitoneal administration of pilocarpine hydrochloride (Merck, SP, Brazil; 320 mg/kg) dissolved in 0.9% sterile saline. Hemopressin, NFKF, rimonabant, and cannabidiol (kindly provided by Professor Raphael Mechoulam, Hebrew University of Jerusalem, Israel, to Professor Francisco Guimarães, Ribeirão Preto Medical School, University of São Paulo, Ribeirão Preto, SP, Brazil) were administered 10 min prior to the administration of pilocarpine. Time to salivation, time to the first motor seizure, number of seizure events, and death were quantified from 0 to 30 min after pilocarpine injection, as previously described [102]. (**E**) Hemopressin or NFKF (1 mg/mL, 500 µL) were freshly prepared in sterile water, or incubated at −20 °C for 24 h or at 100 °C for 15 min, and the peptides that remained in solution were compared by high performance liquid chromatography (HPLC), as previously described [103]. All biological results are expressed as the means ± standard error of the mean (SEM). The statistical comparisons were performed using Student’s *t*-test or analysis of variance (ANOVA), followed by ad-hoc Tukey’s test. Probability less than 0.05 was considered as statistically significant (*p* < 0.05). * *p* < 0.05 vs. control, # *p* < 0.05 vs. HP, ** *p* < 0.05 vs. control, *** *p* < 0.001 vs. control. Data were statistically analyzed with GraphPad Prism software (GraphPad Software Inc, San Diego, CA, USA). (**F**–**I)** Molecular docking studies using the crystallographic structure of CB1R available at the Protein Data Bank (5TGZ). NFKF (panel **I**) has higher binding affinity (higher Goldscore) for CB1R than AM6538 (panel **F**), cannabidiol (**G**) and rimonabant panel (**H**).

Previous studies have suggested that cannabidiol, Δ9-tetrahydrocannabinol (THC), and marijuana have antiepileptic effects, and could be used as adjunctive therapy for epilepsy patients [92,93,94,95,96]. The anticonvulsive effect of Δ9-THC and the CB1R agonist WIN55,212-2 was also shown in models of electroshock-induced generalized convulsive crisis, and pharmacological effects were reversed by administration of CB1R antagonist SR141716A [97,98]. Hemopressin and its fragment NFKF were shown herein to be orally active delaying the first salivation and seizure of pilocarpine-induced seizures in mice (Figure 1A,B). Orally administered NFKF is hundred times more potent than cannabidiol in delaying the first seizure induced by pilocarpine in mice (Figure 1C). Orally administered NFKF is also more efficient in protecting mice from death after pilocarpine-induced seizures (Figure 1D). NFKF has the advantage of being more functionally stable than hemopressin after freezing and heating (Figure 1E). Molecular docking studies using the crystallographic structure of CB1R available at the Protein Data Bank (5TGZ), and selection of the best scoring function of the Gold Program [99,100,101], suggested that NFKF has a higher binding affinity for CB1R than AM6538, cannabidiol, and rimonabant (Figure 1F–I; corresponding Goldscore). Allosteric binding of NFKF to CB1R was suggested by *in vitro* binding assays (data not shown). *In vivo*, orally administrated NFKF-derived sequences NFK, FKL, NF, FK, KF, or KL showed no pharmacological activity in preventing or altering seizures and its symptoms in pilocarpine-induced mice model, whereas NFKL has pharmacological effects similar to that of NFKF (data not shown).

Recently, several conformation-sensitive antibodies targeting G-protein coupled receptors [104,105] were used to screen for novel pharmacologically active intracellular peptides. These screenings identified a novel peptide, DIIADDEPLT (Pep19), with inverse agonist activity at CB1R; Pep19 is derived from a natural intracellular peptide [106]. Oral administration of Pep19 to diet-induced obese Wistar rats significantly reduced adiposity index, whole body weight, glucose, triacylglycerol, cholesterol, and blood pressure, without altering heart rate. In addition, Pep19 oral treatment increased the number of uncoupling-protein 1 (UCP1)-immunostained cells and increased the number and reduced the size of inguinal adipocytes when compared with saline or other treatments. Pep19 also increased UCP1 expression in 3T3-L1 differentiated adipocytes and activated pERK1/2 and AKT signaling pathways. UCP1 expression induced by Pep19 in 3T3-L1 differentiated adipocytes can be blocked by AM251, a CB1R antagonist. Pep19 has no central nervous system effects, as suggested by the lack of brain c-Fos expression, cell toxicity, induction of the cannabinoid tetrad, depressive- and anxiety-like behaviors [88].

It seems worth to mention that in addition to plasma membrane localization of CB1R, it also localizes inside the cells at the membranes of neuronal mitochondria [87]. Through activation of mitochondrial CB1R, hemopressins and other intracellular peptides with affinity for CB1R, such as Pep19-precursor peptide DITADDEPLT [33,88], could modulate cyclic AMP concentration, protein kinase A activity, and mitochondrial function, similar to what have been shown for lipid-derived endocannabinoids. Note that intracellular peptides with endocannabinoid activity are hydrophilic, and co-exist at least in some neuronal populations with hydrophobic endocannabinoids [38]. Therefore, it is possible that intracellular peptides with CB1R activity could function as hydrophilic endocannabinoids from the cytosol to activate mitochondrial CB1R directly, co-modulating with hydrophobic endocannabinoids neuronal energy metabolism [87].

Furthermore, using the substrate-capture assay combined with isotopic-labeling and electron spray mass spectrometry, a new bioactive fragment derived from hemoglobin called AGH (AGHLDDLPGASAL) was identified and pharmacologically characterized [75]. AGH inhibits peripheral hypernociception responses, preferably through μ-type opioid receptors. Although AGH is derived from hemoglobin and has opioid activity, it lacks the key sequence of hemorphins (YPWT), indicating that it may belong to a new class of opioid peptides derived from hemoglobin [75].

## 5. Pharmacological and Biochemical Analyses of Intracellular Peptides Suggest to Function within Cells

Investigating the functionality of intracellular peptides within cells, Cunha et al. [71] used the substrate-capture assay and identified a new group of intracellular peptides. These intracellular peptides were chemically synthesized and artificially reintroduced into the intracellular environment using a covalently bound cell-penetrating peptide. The effects of these intracellular peptides reintroduced into the cells were observed to positively modulate G-protein-coupled receptor (GPCR) signal transduction in CHO and HEK293 cells using a luciferase gene report assay; these intracellular peptides were inactive if administered in the extracellular medium without previous covalent-coupling to the cell-penetrating peptide [71]. Either THOP1 overexpression [71] or siRNA inhibition in HEK293T cells [83] causes change in GPCR signal transduction of both isoproterenol and angiotensin II agonists; cells overexpressing THOP1 respond less to these agonists, while cells with inhibited THOP1 have an increase in signal transduction that was shown to be mediated by protein kinase A [71,83]. Taken together, these studies suggest that intracellular peptides and THOP1 modulate signal transduction of GPCR agonists [71,83]. Since oligopeptidases have substrate-size structural restrictions and metabolize peptides only, one possibility to explain these results is that the intracellular peptide substrates and/or products of THOP1 can alter protein interaction networks affecting the signal transduction of GPCRs. Indeed, intracellular peptides FE2 and FE3, which potentiate the signal transduction of angiotensin and isoproterenol in CHO-S and HEK293T cells, when covalently immobilized on affinity columns interacted with specific proteins involved in signal transduction including dynamin and 14-3-3 [71]. Thus, indirect evidence suggests that intracellular peptides can modulate protein interaction, which results in GPCR signal transduction activation.

Using the *in vitro* technique of surface plasmon resonance, the effect of various intracellular peptides was analyzed on protein interactions related to THOP1, calmodulin (CAM) or 14-3-3ε. At concentrations of 1–50 μM, most of the intracellular peptides tested, including FE2 and FE3, modulated brain cytoplasm protein interactions with CAM or 14-3-3ε. One of these intracellular peptides (VFDVELL; VFD7) that markedly altered the interaction of THOP1 and rat brain proteins with both CAM and 14-3-3ε when introduced into HEK293T cells raised the concentration of cytosolic calcium. The intracellular concentration of this peptide VFD7 in HEK293 cells was estimated to be 16 μM, suggesting its possible biological function in intracellular protein–protein interaction and calcium homeostasis [83]. Therefore, acting on protein–protein interactions may be one of the main mechanisms through which intracellular peptides have biological significance [83].

### 5.1. EL28

The proteasome is an ATP-dependent proteolytic complex consisting of the 19S regulatory particle, which is responsible for recognizing, deploying, and routing the protein into the 20S complex in which the regulatory particle (19S) is attached at both ends [9,107,108,109,110,111]. The 20S complex consists of four heptameric rings, two outer rings (α), and two inner rings (β) [109]. The catalytic subunits of the proteasome are β1, β2, and β5, and have different cleavage specificities in the substrate. The β1 subunit cleaves after acid residues (caspase-like), the β2 subunit cleaves after basic residues (trypsin-like), and the β5 subunit cleaves after hydrophobic residues (chymotrypsin-like). Following proteolytic cleavage, the proteasome releases in the nucleus and cytoplasm peptides containing 2–20 amino acid residues, which may be degraded to amino acids, perform cell signaling functions and/or be presented as antigens via MHC-I [111,112]. Under stress conditions or acute immune response, an alternative form of the proteasome can be induced and the β1, β2, and β5 subunits are replaced by β1i, β2i, and β5i, altering the catalytic specificity and providing better presentation of antigens by MHC-I by the immune proteasome [112]. This change in catalytic specificity that occurs between the constitutive and the immune proteasome at least partially alters the content of various antigenic peptides.

After induction of the immune proteasome in interferon-gamma (INF-γ)-treated HeLa cells, the intracellular peptide repertoire was compared to that of control cells, using semi-quantitative electron spray mass spectrometry [79]. Forty-two peptides were identified, and only one (called EL28) had a threefold increase in relation to the control [79]. EL28 was characterized as a degradation product from proteasome 26S protease regulatory subunit 4 (Rpt2), which is one of the six ATPases present in the 19S regulatory particle of the proteasome [113,114]. When analyzing the effect of the peptide EL28 *in vitro* and *in vivo* on the activity of the proteasome, there was an increase of the caspase-, trypsin-, and chymotrypsin-like activities of the proteasome. The biological significance of EL28 was assessed by measuring its effects on CD8+ T cell proliferation. Using murine splenocytes of C57BL/6JOT1 (OVA-specific CD8^+^ T cells), there is an increase in CD8+ T cell proliferation after treatment with EL28 (100 µM) compared with controls [79,115]. Therefore, EL28 is an intracellular peptide derived from Rpt2 ATPase whose concentration increases in HeLa cells treated with INF-γ. EL28 was reintroduced into cells covalently bound to a cell-penetrating peptide (EL28-cpp) and specifically induced the overexpression of proteasome β5i subunit thereby increasing CD8+ T cell proliferation. Moreover, EL28-cpp was shown to improve and positively modulate the secondary IgG anti-bovine serum albumin immune responsiveness elicited in high antibody-responder mice [79].

### 5.2. PepH

PepH is an intracellular peptide identified in postmortem samples of schizophrenia (SCZ) patients [30]. Histone H2B type 1-H that gives rise to PepH is located in the 8-Mb xMHC region on chromosome 6 that contains over 250 protein-coding genes. Chromosome 6 constitutes about 6% of the human genome according to the “Wellcome Trust Sanger Institute”. The xMHC contains, according to the GWAS (genome-wide association studies), the most significant association for schizophrenia. There are previous reports of histone expression dysregulation in schizophrenia, Huntington’s disease, and autism versus controls cases [116]. PepH contains the highly functional *AVTKY* motif of histone 2B C-terminus [117]. PepH at 50 μM increased Neuro2A cell survival by almost 200% in comparison with the control peptide. Moreover, Neuro2A cells treatment with PepH at 10 μM and 50 μM protected the cells from the toxic effects of LPS. Thus, PepH is an intracellular peptide with cytoprotective effects, suggesting exciting possibilities for its pharmacological use as a modulator of inflammation and oxidative stress responses repeatedly reported in the onset and progression of schizophrenia. Further experiments are necessary to clarify the mechanism of action and the function of PepH in SCZ patients. However, it is possible that PepH participate in structural heterochromatin reorganization/compaction, changing cell survival and protecting Neuro2A cells from LPS toxicity. These findings add evidence to previously described works suggesting the biological significance and functional activity of intracellular peptides in humans.

### 5.3. Pep5

The division cycle of a cell consists of two processes, DNA replication and chromosome segregation to the two nascent cells. This cell division is an alternation between two major stages: (1) mitosis (M), when chromosome condensation, nuclear membrane disassembly, chromatid migration, and separation in two daughter cells takes place, and (2) interphase, which is composed of two gap phases, G1 and G2, and a phase dedicated to DNA synthesis, the S phase [118]. The regulation of the phase transition and the progress through the cell cycle is performed by several complexes of kinases and cyclins. Cyclins are regulatory proteins produced and degraded throughout each phase of the cell cycle that bind to their respective phase-specific partners, cyclin-dependent serine/threonine kinases (CDKs), forming the cyclin-CDK complexes [119,120]. In mammals, there are four major types of cyclins–A, B, D, and E. To perform their roles, cyclins must transit from cytoplasm to nucleus where they can be degraded by the proteasomes; examples include cyclins D and E that translocate to the nucleus to regulate gene transcription [121,122,123]. D-type cyclins (D1, D2 and D3) are expressed in a wide variety of tissues and cell types and are the main allosteric regulators of CDK4 and CDK6 to coordinate cell cycle progression from G1 to S phase. Cyclin D1 is ubiquitously expressed in most cells [124] and more frequently dysregulated than cyclins D2 and D3 in human cancers [125]. On the other hand, cyclin D2 is more tissue-specific and equally related to cancer phenotypes. However, a dual activity has been attributed to cyclin D2 as an oncogene or a tumor suppressor, since its expression can vary depending on the cancer tissues [125].

The cell cycle can also be controlled through proteolysis, for instance, mediating the degradation of cyclins and other biomolecules by the UPS [2,126]. During the cell cycle, two complexes are responsible for poly-ubiquitination of substrates, SCF (Skp1/cullin/F-box proteins) complexes, and APC/C (anaphase promoter complex/cyclosome) [127,128]. Cyclin D1 levels increase during the onset of G1 phase and remain high until the G1/S transition, when it declines [129]. Cyclin D1 degradation is essential for the cells to enter the DNA synthesis phase, and its overexpression in fibroblasts prevents entry of these cells into the S phase [130]. The cell cycle is also controlled by CDKs inhibitory proteins, also known as CKIs, including p21, p27, and p53, whose activities and half-lives are also controlled by SCF-dependent degradation [131].

**Figure 2:**
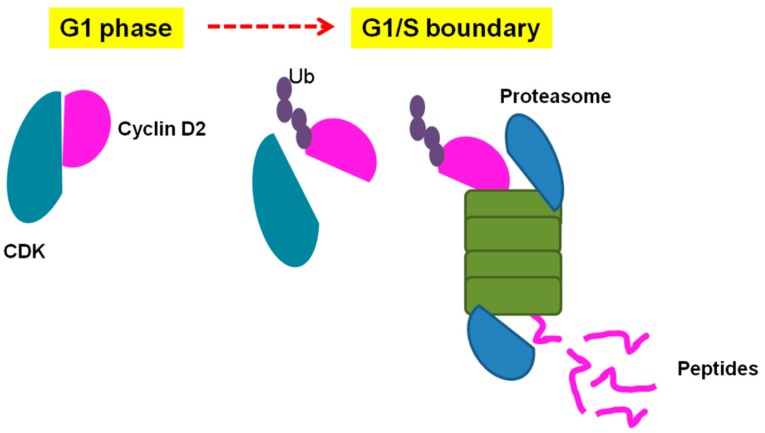
Hypothetical model of Pep5 generation by the ubiquitin–proteasome system (UPS). During G1 phase, cyclin D2 forms a complex with cyclin-dependent kinases (CDKs) to regulate several processes. At the G1/S boundary, the cyclin D2 levels decrease after ubiquitination (Ub) and proteasome degradation. As a result of this degradation, several peptides are formed and released in the intracellular environment. Some of the peptides will be entirely degraded by peptidases whereas some peptides (e.g., Pep5) will remain in the cells to participate in biological processes.

**Figure 3:**
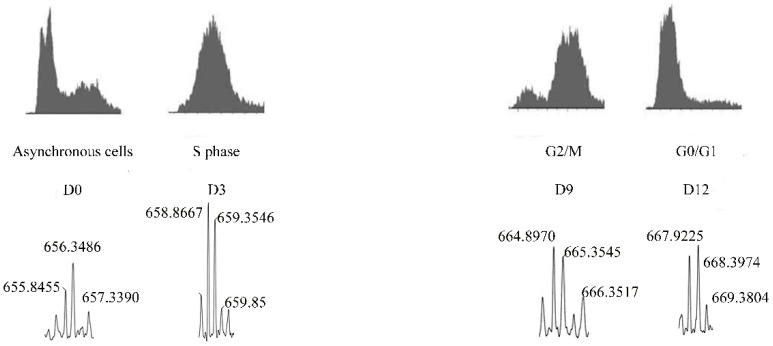
Schematic representation of isotopic-labeling of peptide-containing cellular extracts isolated in different phases of the cell cycle of the Hela cell line. Peptides were extracted from the asynchronous cells and labeled with D0-TMAB, whereas those synchronized in S, G2/M, and G0/G1 were, respectively, labeled with D3-TMAB, D9-TMAB, or D12-TMAB.

Therefore, distinctive intracellular peptides could be produced during specific phases of the cell cycle, considering, for instance, the phase-dependent degradation of cyclins and other molecules, and this could be relevant for progression and/or control of cell division (Figure 2). To investigate a possible relationship between generation of intracellular peptides and cell cycle progression, HeLa cells were used in a well-defined strategy. This human cell line has been extensively used to investigate different molecular aspects of cell cycle control. HeLa cells were synchronized using the double-thymidine block protocol, and intracellular peptides were collected at S, G2/M, and G0/G1, and also from asynchronous control cells. Using isotope labeling and electron spray mass spectrometry experiments, it was possible to compare the relative levels of the different peptides identified in these time periods (Figure 3). A total of 19 peptides, named pep1 through pep19 accordingly, were quantified and sequenced. Among these intracellular peptides, two (pep3, AKADGIVSKNF, a fragment from 40 S ribosomal protein S21; and, Pep5, WELVVLGKL, a fragment of G1/S-specific cyclin D2) varied in specific cell cycle phases. These peptides were chemically synthesized either free or covalently bound to a cell penetrating peptide (cpp, YGRKKRRQRRR) at their C- or N-terminus. This cpp peptide was necessary to allow cell penetration and consequent investigation of the intracellular function of these peptides [40,41]. The initial pharmacological properties of these peptides in cell proliferation and cell death were evaluated in HeLa cells.

In these initial pharmacological experiments, Pep5-cpp has shown to cause extensive cell death in HeLa cells, unlike Pep3 and additional control peptides; neither the other peptides nor the Pep5 without the cell-penetrating peptide covalently coupled changed cell cycle progression or induced cell death [76,77]. Further experiments indicated a strong structure–activity relationship of Pep5 dependent on the tryptophan located at the N-terminus and on the two internal leucines. Thus, the minimal pharmacologically active sequence of Pep5 was determined as WELVVL with the cell-penetrating peptide covalently bound to its C-terminus (WELVVLYGRKKRRQRRR). This minimal Pep5-cpp sequence was also efficient to induce cell death *in vivo*, decreasing the rat C6 glioblastoma tumor volume by approximately 50% after 14 days of treatment. The *in vivo* treatment with Pep5-cpp was conducted during two weeks, using osmotic mini-pumps for constant infusion at the tumor site of 0.5 μL/h of a solution containing 100 μM of either Pep5-cpp or control peptide, diluted in artificial cerebrospinal fluid [76].

Further analyses demonstrated that Pep5-cpp, in concentrations from 50–100 µM, was able to increase cell death in several asynchronous tumor cell lines, such as HeLa, SKRB (human breast cancer cell line), SK-MEL-28 (human skin melanoma cell line), and a rat glial tumor cell line (C6), among others. Some cells seem to be even more sensitive to the pharmacological effects of Pep5-ccp, such as human breast adenocarcinoma cell lines MDA-MB-231 and MCF-7. Normal human thyroid cell line (Nthy-ori 3-1) treated with Pep5-cpp shows a higher survival ratio, suggesting that Pep5 is more effective in tumor cells than in normal cells. More interestingly, MDA-MB-231 cells synchronized in G1/S transition or S phase are more sensitive to Pep5-cpp cell death, which could be associated with the availability of specific targets for the Pep5 in these specific moments of cell cycle [77]. 

**Figure 4:**
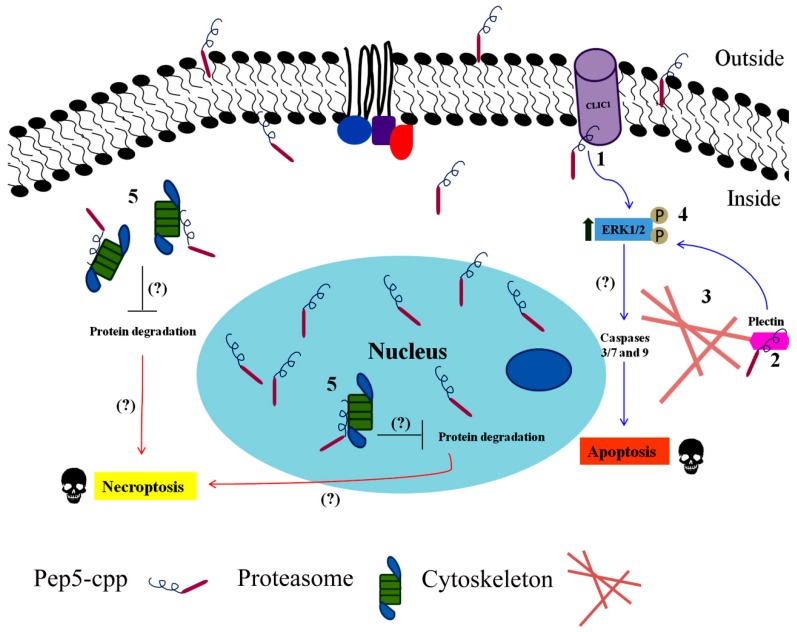
Pep5-cpp leading to cell death seems a combination of events. Once Pep5 is bound to a cell-penetrating peptide, it will carry the cyclin D2 fragment to intracellular compartments such as the cytoplasm and nucleus. (1) Pep5-cpp binds to chloride intracellular channel protein 1 (CLIC1) and (2) Plectin, leading to (3) actin cytoskeleton disorganization and (4) ERK1/2 phosphorylation. The sustained increase in ERK1/2 phosphorylation may contribute to the activation of caspases 3/7 and 9, culminating in the induction of apoptosis. On the other hand, Pep5-cpp also seems to bind to (5) proteasome components causing its inhibition, which in turn can cause protein accumulation in both the cytosol and nucleus, generating signals that will induce programmed necrosis (necroptosis).

An overview of all these results together led us to assume that the cell death caused by Pep5-cpp was likely to be due to a combination of events (Figure 4). Pep5-cpp induces apoptosis through activation of caspases 3/7 and caspase 9 and increases phosphorylation of six proteins related to the MAPK pathway involved in survival and apoptosis induction, such as Akt2, p38, and ERK1/2. This peptide also promotes necrosis, as seen by the cell population labeled with annexin V and propidium iodide. These data were corroborated by pharmacological treatments using distinctive inhibitors of cell death. A combination of necrostatin-1, an inhibitor of necroptosis [132], and qVD, a potent caspase inhibitor, protects cells from death by Pep5-cpp [133]. IM-54, a selective inhibitor of oxidative stress-induced necrosis [134] also reduced the cell death induction caused by Pep5-cpp. Additionally, Pep5-cpp or Pep5 inhibited the beta-5 subunit of the proteasome both *in vivo* and *in vitro*, respectively.

Potential Pep5 targets were investigated in extracts from MDA-MB-231 cells asynchronous or synchronized at G1/S or S phase, using affinity chromatography with covalently immobilized Pep5 followed by electron spray mass spectrometry; empty columns and the cell-penetrating peptide alone were used as controls. Approximately, thirty proteins from each of the experimental conditions evaluated in MDA-MB-231 (i.e., asynchronous or synchronized at G1/S or S phase) were shown to specifically interact with Pep5. Among these proteins, only two proteins were observed to interact with Pep5 in all the experimental conditions evaluated—chloride intracellular channel protein 1 (CLIC1) and plectin [77]. These two proteins are both involved in apoptotic cell death and seem to be relevant to the pharmacological activity of Pep5, as it was shown to induce cell death independent of the phase of the cell cycle.

CLIC is a family of ion channel proteins formed by six cytosolic proteins (CLIC1-6) regularly found soluble or localized in the inner membrane of cells, functionally related to apoptosis and cell cycle regulation [135,136,137]. CLIC1 inhibition increases reactive oxygen species culminating in apoptosis induction through activation of phospho-ERK1/2 in both colon cancer cells and prostate cancer cells [138,139]. The interaction of Pep5 with CLIC1 would be feasible as Pep5-cpp was seen to induce a sustained activation of phospho-ERK1/2 that lasts for at least four hours [77].

Plectin is a member of the plakin family implicated in organizing actin filaments and anchoring the cytoskeletal system to cell junctions. Plectin can be used as a cancer biomarker, since its presence is involved in cell proliferation, migration, and invasion due to its known ability to bind different cytoskeletal components such as tubulin, actin, keratin, and vimentin [140,141,142,143,144,145]. Indeed, Pep5-cpp was seen by confocal microscopy to disorganize actin stress fibers in the MDA-MB-231 cells stained with AlexaFluor 488-labeled phalloidin [77]. Therefore, it is possible that Pep5 induction of apoptosis is mediated at least in part, through the inhibition of CLIC1 and plectin, somehow preventing them from exerting their normal functions inside the cell. Pep5-cpp inhibited the proteasome activity *in vitro* and in addition, several proteins related to the proteasome complex were identified associated with Pep5. This suggests that part of the cell death induced by Pep5-cpp may be through inhibition of the proteasome. It is known that inhibition of proteasome complex by heme proteins causes high oxidative stress and accumulation of damage proteins leading to a programmed necrosis induction in macrophages [146,147].

In view of what was shown above, we believe that Pep5 can promote the activation of cell death in different cell lines via a combination of apoptosis and programmed necrosis. This hypothesis is sustained by using necrostatin-1, a well-known inhibitor of necroptosis (a programmed form of necrosis), combined with inhibitors of caspase-dependent apoptosis, which abolished the cell death effects of Pep5-cpp in HeLa cells.

Recently, Pep5-cpp has been shown to induce cell death in epimastigotes, trypomastigotes, and amastigote forms of *Trypanosoma cruzi,* a parasite vector that is responsible for Chagas disease which is a neglected disease that occurs mainly in the Americas, being considered an important public health issue. Pep5-cpp at lower doses was able to decrease the percentage of infected cells without causing any detectable toxic effects in mammalian host cells. The infective form of *T. cruzi*, i.e., trypomastigotes, pre-treated with Pep5-cpp was unable to infect LLC-MK2 cells. These data suggest that Pep5 can be used as a novel alternative for the treatment of Chagas disease [78]. Altogether, these findings highlight the therapeutic potential of Pep5.

## 6. Perspectives

The initial and intriguing question that raised our interest in investigating intracellular peptides biology and pharmacology, was that only one peptide from each cellular protein was supposedly to escape degradation to be presented to the immune system associated to MHC-I. Hypothetically, if every cell can express one peptide from its ~10,000 different proteins, and each protein has in average 500 amino acids, 500,000 different peptides can be potentially generated by UPS in every cell considering that the average size for proteasome products are peptides of 10 amino acids. Based on these initial questionings, we started investigating if these additional intracellular peptides could be natural substrates of oligopeptidases THOP1 and/or Nln within cells. These initial studies on intracellular peptides became a broad field for investigation, with many unanswered questions. Some of these unanswered questions include (1) the mechanism of action of intracellular peptides, (2) the enzymes participating in their biosynthetic and degradative pathways, (3) the secretory pathways, and (4) how intracellular peptides escape complete degradation. Because the majority of intracellular peptides were shown to remain inside the cells [54], it is possible that their main biological functions occur within cells. However, a significant portion of intracellular peptides can be secreted and many can be functional, binding to cell surface receptors similar to neuropeptides.

In general, peptides are selective and effective signaling molecules that bind to extracellular sites of specific plasma membrane receptors, such as GPCRs, enzyme-linked receptors, integrins, or ion channels, where they trigger signal transduction. Given their attractive pharmacological profile and intrinsic properties, peptides represent an excellent starting point for planning new therapeutics, and their specificity and usually low toxicity, translates into excellent safety, tolerability, and efficacy profiles for human administration. However, the short half-life and the immunogenic potential of peptides seems to limit the interest of many pharmaceutical industries for peptide-based new drugs. Intracellular peptides are produced by limited proteolysis from a specific set of intracellular proteins, and within cells must have been selected for their increased proteolytic stability. Therefore, screening intracellular peptides for new peptide-based drug discovery could be advantageous at least in the number of hits to test, compared to screenings using peptide libraries or cryptides from enzymatic digestion of different protein sources. Some advantages of using intracellular peptides to screen for potential drugs could be (1) smaller number of peptides to start screening for pharmacological activity (e.g., Pep19); (2) possibility to choose the intracellular peptides to screening after challenging cells with a stimulus of interest (e.g., Pep5 and EL28); (3) possibility to find functional intracellular peptides that are differentially present when comparing health and disease states of patients (e.g., PepH); (4) possibility to find functional intracellular peptides comparing different animal models (e.g., wild-type C57BL6 versus knockout mice). Hemopressin, NFKF, and Pep19 were also used herein as examples of intracellular peptides with the advantage of been orally active. Oral administration of intracellular peptides should be relevant to reduce their potential to elicit immune response after chronic administration.

Changes in the intracellular peptidome following inhibition of THOP1 expression by siRNA altered the relative concentration of specific intracellular peptides, and in parallel caused a protein-kinase A-mediated potentiation of isoproterenol signal transduction in HEK293 cells [83]. An increase in specific intracellular peptides in gastrocnemius and epididymal adipose tissues correlated with an increased glucose tolerance and insulin sensitivity of Nln^−/−^ [84]. Therefore, changes in the intracellular peptidome correlates with changes in cell signal transduction and animal physiology, suggesting that intracellular peptides are biologically significant.

Delivery of intracellular peptides within cells is challenging and novel technological developments for *in vivo* peptide-drug delivery systems are still necessary. One possibility is that intracellular peptides bound to a cell-penetrating peptide could be administrated directly inside tumors [76]. Other possibilities that need further investigation are the use of fusion proteins to deliver and internalize peptides to specific tissues, peptide-carrier systems like liposomes or nanoparticles, and the use of viral vectors. It is important to mention that a rationally-designed retroinverse peptide that inhibits the nuclear interaction of FOXO4 and p53, was capable of neutralizing doxorubicin-induced chemotoxicity and restored fitness and renal function in both fast aging XpdTTD/TTD and naturally aged mice [148]. Moreover, another rationally-designed peptide (SAMβA) that selectively antagonizes intracellular Mfn1–βIIPKC association, protects cultured neonatal and adult cardiac myocytes, and re-establishes mitochondrial morphology and function and improves cardiac contractility in rats with heart failure. These results suggest that SAMβA may be a peptide-based drug for treatment of patients with heart failure [149].

Taken altogether, intracellular peptides seem an exciting area for additional scientific investigations. The biological significance of intracellular peptides could be broad, modulating cell signaling from inside and outside the cells. Intracellular peptides could also be interesting prototypes for developing novel therapeutically efficient peptide-based drugs.

## Figures and Tables

**Table 1:** Intracellular peptides with characterized pharmacological activities.

Peptide Name	Amino Acid Sequence	Protein Precursor	Pharmacological Activity	Level of Evidence	Reference(s)
Hemopressin	PVNFKFLSH	Hemoglobin alpha-chain	First intracellular peptide identified using the substrate-capture assay. Has hypotensive action in anesthetized rats if administered intravenously or intra arterially. Was found to bind CB1R receptor as an inverse agonist and to have oral activity in rats and mice with antinociceptive action in hyperalgesia models. Also, orally administrated is capable to reduce appetite in experimental rat and mouse models. It has potent activity inducing myelination.	Bind CB1R receptor as inverse agonist (EC50 = 0.35 nM); increases adenylyl cyclase activity in rat striatal membranes. The short hemopressin sequence PVNFKF was shown to have inverse agonist activity on CB1R receptors.	[14,25,33,34,35,36,37,38,39,40,41,42,43,44,45,46,47,48,49,50,51,52,53,54,55,56,57,58,59,60,61,62,63,64,65,66,67,68,69,70]
VD and RVD-hemopressin	RVDPVNFKFLSH	Hemoglobin alpha-chain	Found as the endogenous hemopressins, have CB1R receptor agonist activity in opposition to inverse agonist activity of hemopressin. Also described as negative allosteric modulator of CB1R receptors.	Found in mouse blood. Increase cannabinoid 1 and 2 receptor-mediated intracellular Ca^2+^ levels in HEK-293 cells; effect is blocked by SR141716. Induce neurite outgrowth in Neuro 2A cells. Several variants of RVD-hemopressin retain CB1R pharmacological activity.	[35,36,37]
NFKF	NFKF	Hemoglobin alpha-chain	Hemopressin (PVNFKFLSH; HP) and its smallest CB1R active fragment NFKF are both orally active, and delays symptoms and seizures of pilocarpine-induced seizures in mice. Orally administrated NFKF is 100 times more potent than cannabidiol in delaying the first seizure induced by pilocarpine in mice. Orally administrated NFKF is more efficient in protecting mice from death after pilocarpine-induced seizures. NFKF has the advantage of being more functionally stable than hemopressin after freezing and heating.	Molecular docking suggests that NFKF has a better Goldscore for binding to CB1R than AM6538, cannabidiol, and rimonabant. *In vivo* assays show that orally administrated NFKF is very efficient in preventing seizures and its symptoms in pilocarpine-induced mice model. NFKF administered orally is a potent cannabinoid for treating epilepsy seizures and has economic advantages over cannabidiol use. *In vivo* assays show that orally administrated NFKF-derived sequence NFKL has similar properties compared to NFKF, whereas NFK, FKL, NF, FK, KF, or KL shown no pharmacological activity in preventing or altering seizures and its symptoms in pilocarpine-induced mice model (data not shown).	Original data, presented herein.
Pep19	DIIADDEPLT	None (synthetic non-natural peptide)	The original intracellular peptide is derived from peptidyl-prolyl cis–trans isomerase A (DITADDEPLT), and was rationally modified in specific amino acids to generate pep19 (DIIADDEPLT), which, compared to the natural intracellular peptide, shows a better inverse agonist activity binding to CB1R receptors, with a lack of undesired CNS effects. Changes in Pep19 amino acid sequence strongly affect its specificity and CB1R pharmacological properties. Pep19-induced uncoupling-protein 1 expression in both white adipose tissue and 3T3-L1 differentiated adipocytes activates pERK1/2 and AKT signaling pathways. Uncoupling-protein 1 expression induced by Pep19 in 3T3-L1 differentiated adipocytes is blocked by AM251, a CB1R receptor antagonist.	*In vivo* and *in vitro* inverse agonist of CB1R receptors; has the pharmacological advantage of not having undesired CNS cannabinoid activity; bind CB1R receptor as inverse agonist (EC50 = 0.49 nM); orally administrated in rats reduces adiposity index and body weight, and improves several metabolic parameters including reduction in the glucose, triacylglycerol, cholesterol, and blood pressure, without altering heart rate in obese rats.	[35,36]; Patent granted in USA (US9796760) and Europe (EP2878306).
FE2	PGANAAAAKIQASFR	Neurogranin	Modulates AT1 and β1/2-adrenergic G-protein coupled receptors signal transduction in CHO and HEK293 cells. The mechanism of action likely involves competition to protein kinase C’s natural substrates, and binding to specific proteins or protein complex including dynamin 1, alpha-adaptin A2, alpha1- and beta2c-tubulin, vesicular fusion protein NSF, Rab GDP dissociation inhibitor, and several 14-3-3 isoforms.	Only if coupled to cell-penetrating peptide through a Cys–Cys bond that dissociate from the intracellular peptide upon internalization in HEK293 and CHO-S cells, this peptide at 80 µM concentration potentiates both angiotensin II and isoproterenol agonist action. The high concentration needed for pharmacological activity is probably due to the high degradation ratio of the free intracellular peptide, after it is released from cell-penetrating peptide into the cytosol.	[71]
FE3	SSGAHGEEGSARIWKA	Cytochrome-c oxidase	Modulates AT1 and β1/2-adrenergic G-protein coupled receptors’ signal transduction in CHO and HEK293 cells. The mechanism of action is not related to competition to protein kinase C, whereas it binds to specific proteins or protein complex including dynamin 1, alpha1- and beta-tubulin, vesicular fusion protein NSF, amphyphisin 1, and alpha-adaptin C. It was observed to increase the interaction of both calmodulin and 14-3-3 epsilon with mice brain proteins.	Only if coupled to cell-penetrating peptide through a Cys–Cys bond that dissociate from the intracellular peptide upon internalization in HEK293 and CHO-S cells, this peptide at 80 µM concentration potentiates both angiotensin II and isoproterenol agonist action. The high concentration needed for pharmacological activity is probably due to the high degradation ratio of the free intracellular peptide, after it is released from cell-penetrating peptide into the cytosol.	[71,72]
DBI	TVGDVNTDRPGLLDL	Acyl-CoA-binding protein	Intracellular peptide originally described as an agonist of benzodiazepine receptors and termed “diazepam-binding inhibitor” (DBI); facilitates the transport of glucose stimulated by insulin in 3T3-L1 adipocytes both in regular and insulin-resistant 3T3L1 differentiated adipocytes; binds to heat shock protein 8 only in epididymal adipose tissue extracts obtained from obese rats that were fed a Western diet.	DBI is a competitive inhibitor for the binding of [3H] diazepam to GABA receptors with a Ki of 4 µM concentration. DBI’s relative concentration was found to increase in the epididymal adipose tissue extracted from obese rats that were fed a Western diet, compared to non-obese rats that were fed a control diet. At concentrations of 0.1–1 nM, the peptide potentiated insulin-induced glucose uptake in 3T3-L1 differentiated adipocytes. DBI has no effects in glucose uptake in the absence of insulin or without being cell internalized through transient cell permeabilization with CHAPS 0.1%.	[11,73]
LDBI	GDVNTDRPGLLDL	Acyl-CoA-binding protein	LDBI is a shorter version of DBI lacking the two N-terminal amino acids. It was shown to facilitate glucose transport stimulated by insulin in 3T3-L1 adipocytes, both in regular and insulin-resistant 3T3L1 differentiated adipocytes. In addition to heat shock protein 8, LDBI specifically binds to additional proteins only in epididymal adipose tissue extracted from obese rats, including annexin A6, asporin, ATP synthase H+ transporting mitochondrial F1 complex beta polypeptide isoform CRA_a, complement component 4A, protein 1 (HMG-1), and Ig gamma-2A chain C region.	LDBI’s relative concentration was found to increase in the epididymal adipose tissue extracted from obese rats that were fed a Western diet compared to non-obese rats that were fed a control diet. At concentrations of 0.1–1 nM, the peptide potentiated insulin-induced glucose uptake in 3T3-L1 differentiated adipocytes. LDBI has no effects in glucose uptake in the absence of insulin or without being cell internalized through transient cell permeabilization with CHAPS 0.1%.	[73]
VFD-7	VFDVELL	Peptidyl-prolyl cis–trans isomerase	*In vitro*, using surface plasmon resonance assay, it was found that the peptide inhibits the interaction of calmodulin and 14-3-3 with mice cytoplasmic brain proteins. It strongly inhibits the interaction of recombinant THOP1 with calmodulin at 1 and 10 µM concentrations; however, VFD7 is not able to disrupt this interaction after it is assembled. It stimulates the unconventional secretion of THOP1 at 10 µM concentration. It increases the concentration of Ca^2+^ in a dose-dependent manner starting at 10 µM concentration.	Intracellular VFD-7 quantification using MS with isotope labeling suggest that in HEK293 cells, its intracellular concentration is 16 ± 3 μM. Treatment of HEK293 cells with either 0.2 µM of epoxomicin or carfilzomib 1µM, for 1 h or 35 min, respectively, reduces more than 5 times the concentration of VFD7 in HEK293 cells, which may suggest its participation on clinical benefits obtained with proteasome inhibitors.	[29,72,74]
AGH	AGHLDDLPGALSAL	Hemoglobin alpha-chain	Identified in rat brain homogenates using the substrate-capture assay; inhibits peripheral hyperalgesia response through the activation of opioid receptors.	AGH (10 µ/paw) has peripheral antinociceptive effects on paw carrageenan-induced hyperalgesia in Wistar rats, which was antagonized by naloxone. However, AGH was neither observed to bind opioid receptors nor to have similar opioid analgesic central effects.	[75]
Pep5	WELVVLGKL	Cyclin D2	Identified to increase in G1/S cell cycle of HeLa cells. Only if coupled to a cell-penetrating peptide (Pep5-cpp), the peptide induces cell death in several tumor cells, and *in vivo* reduces 50% of the size of C6 glioblastoma in rat brain. Pep5-cpp activates caspases 3/7 and 9, inhibits the phosphorylation of Akt2, activates p38α and -γ, and inhibits proteasome activity. N-terminal tryptophan removal as well as Leu to Ala substitutions totally abolishes the cell death activity by Pep5-cpp; the minimal pharmacological active sequence is WELVVL. Pep5-cpp also induces cell death in epimastigotes, trypomastigotes, and amastigotes forms of *Trypanosoma cruzi* parasites responsible for Chagas disease. At low doses, Pep5-cpp decreases the percentage of infected cells without any detectable toxic effects in mammalian host cells. The infective form of *T. cruzi*, i.e., trypomastigotes, pre-treated with Pep5-cpp was unable to infect LLC-MK2 cells.	Pep5-cpp (25 µM) cell death was significantly increased when the peptide was added at G1/S or S phases of the cell cycle compared to the effects of the peptide on asynchronous cells. Pep5-cpp treatment caused a major disruption of the stress F-actin fibers’ integrity after only 4 h of treatment at 25 µM. ERK1/2 phosphorylation is increased following pep5-cpp treatment in both asynchronous or synchronized cells; however, if added to cells synchronized in S phase, pep5-cpp induces a significant increase in ERK1/2 phosphorylation that remains high for more than 4 h. In mammalian cells, pep5 binds to different proteins depending of the cell cycle phases; however, at least two proteins, plectin and cytosolic cloride channel (CLIC1), were targeted by pep5 in either asynchronous or synchronized MDA-MB-231 cells. In *Tripanossoma cruzi*, a different set of specific proteins were identified to bind pep5, including calmodulin-ubiquitin-associated protein, GTPase activating protein, and a putative protein kinase.	[76,77,78]
EL28	VGSELIQKY	Human 19S ATPase regulatory subunit 4	Peptide identified after its relative concentration increased in HeLa cells following treatment with gamma interferon. Intracellular peptide activator of immune proteasome and proliferation of CD8+.	*In vitro*, EL28 (50 µM) increased the chymotrypsin, trypsin, and caspase-like proteasome activities. *In vivo* only when linked to a cell-penetrating peptide, EL28 (100µM) potentiated the ability of interferon-gamma to stimulate the expression of the immunoproteasome β5i subunit, and increase the proliferation of CD8+ T-cells. The EL28-cell-penetrating peptide improved and positively modulated the secondary IgG anti-bovine serum albumin immune responsiveness elicited in high antibody-responder mice.	[79]
PepH	SEGTKAVTKYTSSK	Histone H2B	In Neuro2A cells, PepH bound to a cell-penetrating peptide (PepH-cpp, 50 μM) showed a protective effect against cell death. PepH-cpp (10–50 μM) significantly prevented Neuro2A cells death induced by lipopolysaccharide.	Decreased in the anterior temporal lobe of brains of patients with schizophrenia when compared with healthy individuals (postmortem).	[30]

## References

[1] Fricker L.D. (2005). Neuropeptide-processing enzymes: Applications for drug discovery. AAPS J..

[2] Glickman M.H., Ciechanover A. (2002). The ubiquitin-proteasome proteolytic pathway: Destruction for the sake of construction. Physiol. Rev..

[3] Dolan B.P., Bennink J.R., Yewdell J.W. (2011). Translating DRiPs: Progress in understanding viral and cellular sources of MHC class I peptide ligands. Cell. Mol. Life Sci..

[4] Lee C., Zeng J., Drew B.G., Sallam T., Martin-Montalvo A., Wan J., Kim S.J., Mehta H., Hevener A.L., de Cabo R. (2015). The mitochondrial-derived peptide MOTS-c promotes metabolic homeostasis and reduces obesity and insulin resistance. Cell Metab..

[5] Rist M.J., Theodossis A., Croft N.P., Neller M.A., Welland A., Chen Z., Sullivan L.C., Burrows J.M., Miles J.J., Brennan R.M. (2013). HLA peptide length preferences control CD8+ T cell responses. J. Immunol..

[6] Burrows J.M., Bell M.J., Brennan R., Miles J.J., Khanna R., Burrows S.R. (2008). Preferential binding of unusually long peptides to MHC class I and its influence on the selection of target peptides for T cell recognition. Mol. Immunol..

[7] Kloverpris H.N., Stryhn A., Harndahl M., Payne R., Towers G.J., Chen F., Riddell L., Walker B.D., Ndung’u T., Leslie A. (2013). HLA-specific intracellular epitope processing shapes an immunodominance pattern for HLA-B*57 that is distinct from HLA-B*58:01. J. Virol..

[8] Caron E., Kowalewski D.J., Chiek Koh C., Sturm T., Schuster H., Aebersold R. (2015). Analysis of Major Histocompatibility Complex (MHC) Immunopeptidomes Using Mass Spectrometry. Mol. Cell. Proteom..

[9] Connell G.E., Watson R.W. (1957). Intracellular peptides of Pseudomonas hydrophila. Biochim. Biophys. Acta.

[10] McManus I. (1958). Synthesis of intracellular peptides in Torula utilis. J. Biol. Chem..

[11] Guidotti A., Forchetti C.M., Corda M.G., Konkel D., Bennett C.D., Costa E. (1983). Isolation, characterization, and purification to homogeneity of an endogenous polypeptide with agonistic action on benzodiazepine receptors. Proc. Natl. Acad. Sci. USA.

[12] Alho H., Costa E., Ferrero P., Fujimoto M., Cosenza-Murphy D., Guidotti A. (1985). Diazepam-binding inhibitor: A neuropeptide located in selected neuronal populations of rat brain. Science.

[13] Huyer G., Kistler A., Nouvet F.J., George C.M., Boyle M.L., Michaelis S. (2006). Saccharomyces cerevisiae a-factor mutants reveal residues critical for processing, activity, and export. Eukaryot. Cell.

[14] Rioli V., Gozzo F.C., Heimann A.S., Linardi A., Krieger J.E., Shida C.S., Almeida P.C., Hyslop S., Eberlin M.N., Ferro E.S. (2003). Novel natural peptide substrates for endopeptidase 24.15, neurolysin, and angiotensin-converting enzyme. J. Biol. Chem..

[15] Rioli V., Ferro E.S. (2018). Substrate Capture Assay Using Inactive Oligopeptidases to Identify Novel Peptides. Methods Mol. Biol..

[16] Dale C.S., Pagano Rde L., Rioli V. (2005). Hemopressin: A novel bioactive peptide derived from the alpha1-chain of hemoglobin. Mem Inst. Oswaldo Cruz.

[17] Heimann A.S., Favarato M.H., Gozzo F.C., Rioli V., Carreno F.R., Eberlin M.N., Ferro E.S., Krege J.H., Krieger J.E. (2005). ACE gene titration in mice uncovers a new mechanism for ACE on the control of body weight. Physiol. Genom..

[18] Machado M.F., Cunha F.M., Berti D.A., Heimann A.S., Klitzke C.F., Rioli V., Oliveira V., Ferro E.S. (2006). Substrate phosphorylation affects degradation and interaction to endopeptidase 24.15, neurolysin, and angiotensin-converting enzyme. Biochem. Biophys. Res. Commun..

[19] Ferro E.S., Hyslop S., Camargo A.C. (2004). Intracellullar peptides as putative natural regulators of protein interactions. J. Neurochem..

[20] Fesenko I.A., Arapidi G.P., Skripnikov A.Y., Alexeev D.G., Kostryukova E.S., Manolov A.I., Altukhov I.A., Khazigaleeva R.A., Seredina A.V., Kovalchuk S.I. (2015). Specific pools of endogenous peptides are present in gametophore, protonema, and protoplast cells of the moss Physcomitrella patens. BMC Plant. Biol..

[21] Fesenko I., Khazigaleeva R., Govorun V., Ivanov V. (2018). Analysis of Endogenous Peptide Pools of Physcomitrella patens Moss. Methods Mol. Biol..

[22] Dasgupta S., Yang C., Castro L.M., Tashima A.K., Ferro E.S., Moir R.D., Willis I.M., Fricker L.D. (2016). Analysis of the Yeast Peptidome and Comparison with the Human Peptidome. PLoS ONE.

[23] Teixeira C.M.M., Correa C.N., Iwai L.K., Ferro E.S., Castro L.M. (2019). Characterization of intracellular peptides from zebrafish *Danio rerio* brain”. Zebrafish.

[24] Fricker L.D. (2010). Analysis of mouse brain peptides using mass spectrometry-based peptidomics: Implications for novel functions ranging from non-classical neuropeptides to microproteins. Mol. Biosyst..

[25] Gelman J.S., Sironi J., Castro L.M., Ferro E.S., Fricker L.D. (2010). Hemopressins and other hemoglobin-derived peptides in mouse brain: Comparison between brain, blood, and heart peptidome and regulation in Cpefat/fat mice. J. Neurochem..

[26] Berezniuk I., Sironi J., Callaway M.B., Castro L.M., Hirata I.Y., Ferro E.S., Fricker L.D. (2010). CCP1/Nna1 functions in protein turnover in mouse brain: Implications for cell death in Purkinje cell degeneration mice. FASEB J..

[27] Berti D.A., Morano C., Russo L.C., Castro L.M., Cunha F.M., Zhang X., Sironi J., Klitzke C.F., Ferro E.S., Fricker L.D. (2009). Analysis of intracellular substrates and products of thimet oligopeptidase in human embryonic kidney 293 cells. J. Biol. Chem..

[28] Gelman J.S., Sironi J., Castro L.M., Ferro E.S., Fricker L.D. (2011). Peptidomic analysis of human cell lines. J. Proteom. Res..

[29] Dasgupta S., Castro L.M., Dulman R., Yang C., Schmidt M., Ferro E.S., Fricker L.D. (2014). Proteasome inhibitors alter levels of intracellular peptides in HEK293T and SH-SY5Y cells. PLoS ONE.

[30] Cafe-Mendes C.C., Ferro E.S., Torrao A.S., Crunfli F., Rioli V., Schmitt A., Falkai P., Britto L.R., Turck C.W., Martins-de-Souza D. (2017). Peptidomic analysis of the anterior temporal lobe and corpus callosum from schizophrenia patients. J. Proteom..

[31] Li Y., Wang X., Wang F., You L., Xu P., Cao Y., Chen L., Wen J., Guo X., Cui X. (2018). Identification of intracellular peptides associated with thermogenesis in human brown adipocytes. J. Cell. Physiol..

[32] Murata S., Takahama Y., Kasahara M., Tanaka K. (2018). The immunoproteasome and thymoproteasome: Functions, evolution and human disease. Nat. Immunol..

[33] Heimann A.S., Gomes I., Dale C.S., Pagano R.L., Gupta A., de Souza L.L., Luchessi A.D., Castro L.M., Giorgi R., Rioli V. (2007). Hemopressin is an inverse agonist of CB1 cannabinoid receptors. Proc. Natl. Acad. Sci. USA.

[34] Dodd G.T., Mancini G., Lutz B., Luckman S.M. (2010). The peptide hemopressin acts through CB1 cannabinoid receptors to reduce food intake in rats and mice. J. Neurosci..

[35] Gomes I., Grushko J.S., Golebiewska U., Hoogendoorn S., Gupta A., Heimann A.S., Ferro E.S., Scarlata S., Fricker L.D., Devi L.A. (2009). Novel endogenous peptide agonists of cannabinoid receptors. FASEB J..

[36] Gomes I., Dale C.S., Casten K., Geigner M.A., Gozzo F.C., Ferro E.S., Heimann A.S., Devi L.A. (2010). Hemoglobin-derived peptides as novel type of bioactive signaling molecules. AAPS J..

[37] Bauer M., Chicca A., Tamborrini M., Eisen D., Lerner R., Lutz B., Poetz O., Pluschke G., Gertsch J. (2012). Identification and quantification of a new family of peptide endocannabinoids (Pepcans) showing negative allosteric modulation at CB1 receptors. J. Biol. Chem..

[38] Hofer S.C., Ralvenius W.T., Gachet M.S., Fritschy J.M., Zeilhofer H.U., Gertsch J. (2015). Localization and production of peptide endocannabinoids in the rodent CNS and adrenal medulla. Neuropharmacology.

[39] Xapelli S., Agasse F., Grade S., Bernardino L., Ribeiro F.F., Schitine C.S., Heimann A.S., Ferro E.S., Sebastiao A.M., De Melo Reis R.A. (2014). Modulation of subventricular zone oligodendrogenesis: A role for hemopressin?. Front. Cell. Neurosci..

[40] Khilnani G., Khilnani A.K. (2011). Inverse agonism and its therapeutic significance. Indian J. Pharmacol..

[41] Bomar M.G., Samuelsson S.J., Kibler P., Kodukula K., Galande A.K. (2012). Hemopressin forms self-assembled fibrillar nanostructures under physiologically relevant conditions. Biomacromolecules.

[42] Dale C.S., Pagano Rde L., Rioli V., Hyslop S., Giorgi R., Ferro E.S. (2005). Antinociceptive action of hemopressin in experimental hyperalgesia. Peptides.

[43] Blais P.A., Cote J., Morin J., Larouche A., Gendron G., Fortier A., Regoli D., Neugebauer W., Gobeil F. (2005). Hypotensive effects of hemopressin and bradykinin in rabbits, rats and mice. A comparative study. Peptides.

[44] Lippton H., Lin B., Gumusel B., Witriol N., Wasserman A., Knight M. (2006). Hemopressin, a hemoglobin fragment, dilates the rat systemic vascular bed through release of nitric oxide. Peptides.

[45] Gelman J.S., Fricker L.D. (2010). Hemopressin and other bioactive peptides from cytosolic proteins: Are these non-classical neuropeptides?. AAPS J..

[46] Scrima M., Di Marino S., Grimaldi M., Mastrogiacomo A., Novellino E., Bifulco M., D’Ursi A.M. (2010). Binding of the hemopressin peptide to the cannabinoid CB1 receptor: Structural insights. Biochemistry.

[47] Horvath G., Mecs L. (2011). Antinociception by endogenous ligands at peripheral level. Ideggyogy Sz.

[48] Hama A., Sagen J. (2011). Activation of spinal and supraspinal cannabinoid-1 receptors leads to antinociception in a rat model of neuropathic spinal cord injury pain. Brain Res..

[49] Hama A., Sagen J. (2011). Centrally mediated antinociceptive effects of cannabinoid receptor ligands in rat models of nociception. Pharmacol. Biochem. Behav..

[50] Petrovszki Z., Kovacs G., Tomboly C., Benedek G., Horvath G. (2012). The effects of peptide and lipid endocannabinoids on arthritic pain at the spinal level. Anest. Anal.g.

[51] Bomar M.G., Galande A.K. (2013). Modulation of the cannabinoid receptors by hemopressin peptides. Life Sci..

[52] Zhou L., Jin Q., Yang Y., Liu Z., Li X., Dong S., Zhao L. (2012). Effects of endokinin A/B and endokinin C/D on the antinociception properties of hemopressin in mice. Peptides.

[53] Reddy P.A., Jones S.T., Lewin A.H., Carroll F.I. (2012). Synthesis of hemopressin peptides by classical solution phase fragment condensation. Int. J. Pept..

[54] Gelman J.S., Dasgupta S., Berezniuk I., Fricker L.D. (2013). Analysis of peptides secreted from cultured mouse brain tissue. Biochim. Biophys. Acta.

[55] Dodd G.T., Worth A.A., Hodkinson D.J., Srivastava R.K., Lutz B., Williams S.R., Luckman S.M. (2013). Central functional response to the novel peptide cannabinoid, hemopressin. Neuropharmacology.

[56] Tanaka K., Shimizu T., Yanagita T., Nemoto T., Nakamura K., Taniuchi K., Dimitriadis F., Yokotani K., Saito M. (2014). Brain RVD-haemopressin, a haemoglobin-derived peptide, inhibits bombesin-induced central activation of adrenomedullary outflow in the rat. Br. J. Pharmacol..

[57] Rashid M., Wangler N.J., Yang L., Shah K., Arumugam T.V., Abbruscato T.J., Karamyan V.T. (2014). Functional up-regulation of endopeptidase neurolysin during post-acute and early recovery phases of experimental stroke in mouse brain. J. Neurochem..

[58] Han Z.L., Fang Q., Wang Z.L., Li X.H., Li N., Chang X.M., Pan J.X., Tang H.Z., Wang R. (2014). Antinociceptive effects of central administration of the endogenous cannabinoid receptor type 1 agonist VDPVNFKLLSH-OH [(m)VD-hemopressin(alpha)], an N-terminally extended hemopressin peptide. J. Pharmacol. Exp. Ther..

[59] Li X.H., Li N., Wang Z.L., Pan J.X., Han Z.L., Chang X.M., Tang H.H., Wang P., Wang R., Fang Q. (2014). The hypotensive effect of intrathecally injected (m)VD-hemopressin(alpha) in urethane-anesthetized rats. Peptides.

[60] Toniolo E.F., Maique E.T., Ferreira W.A., Heimann A.S., Ferro E.S., Ramos-Ortolaza D.L., Miller L., Devi L.A., Dale C.S. (2014). Hemopressin, an inverse agonist of cannabinoid receptors, inhibits neuropathic pain in rats. Peptides.

[61] Pan J.X., Wang Z.L., Li N., Han Z.L., Li X.H., Tang H.H., Wang P., Zheng T., Fang Q., Wang R. (2014). Analgesic tolerance and cross-tolerance to the cannabinoid receptors ligands hemopressin, VD-hemopressin(alpha) and WIN55,212-2 at the supraspinal level in mice. Neurosci. Lett..

[62] Mahmoud M.F., Swefy S.E., Hasan R.A., Ibrahim A. (2014). Role of cannabinoid receptors in hepatic fibrosis and apoptosis associated with bile duct ligation in rats. Eur. J. Pharmacol..

[63] Fogaca M.V., Sonego A.B., Rioli V., Gozzo F.C., Dale C.S., Ferro E.S., Guimaraes F.S. (2015). Anxiogenic-like effects induced by hemopressin in rats. Pharmacol. Biochem. Behav..

[64] Song B., Kibler P.D., Endsley A.N., Nayak S.K., Galande A.K., Jambunathan K. (2015). Site-specific Substitutions Eliminate Aggregation Properties of Hemopressin. Chem. Biol. Drug Des..

[65] Straiker A., Mitjavila J., Yin D., Gibson A., Mackie K. (2015). Aiming for allosterism: Evaluation of allosteric modulators of CB1 in a neuronal model. Pharmacol. Res..

[66] Ma L., Jia J., Niu W., Jiang T., Zhai Q., Yang L., Bai F., Wang Q., Xiong L. (2015). Mitochondrial CB1 receptor is involved in ACEA-induced protective effects on neurons and mitochondrial functions. Sci. Rep..

[67] Szlavicz E., Perera P.S., Tomboly C., Helyes Z., Zador F., Benyhe S., Borsodi A., Bojnik E. (2015). Further Characterization of Hemopressin Peptide Fragments in the Opioid and Cannabinoid Systems. Anest. Analg..

[68] Zhang L., Kolaj M., Renaud L.P. (2015). Intracellular postsynaptic cannabinoid receptors link thyrotropin-releasing hormone receptors to TRPC-like channels in thalamic paraventricular nucleus neurons. Neuroscience.

[69] Pan J.X., Wang Z.L., Li N., Zhang N., Wang P., Tang H.H., Zhang T., Yu H.P., Zhang R., Zheng T. (2015). Effects of neuropeptide FF and related peptides on the antinociceptive activities of VD-hemopressin(alpha) in naive and cannabinoid-tolerant mice. Eur. J. Pharmacol..

[70] El Swefy S., Hasan R.A., Ibrahim A., Mahmoud M.F. (2015). Curcumin and hemopressin treatment attenuates cholestasis-induced liver fibrosis in rats: Role of CB1 receptors. Naunyn Schmiedebergs Arch. Pharmacol..

[71] Cunha F.M., Berti D.A., Ferreira Z.S., Klitzke C.F., Markus R.P., Ferro E.S. (2008). Intracellular peptides as natural regulators of cell signaling. J. Biol. Chem..

[72] Russo L.C., Asega A.F., Castro L.M., Negraes P.D., Cruz L., Gozzo F.C., Ulrich H., Camargo A.C., Rioli V., Ferro E.S. (2012). Natural intracellular peptides can modulate the interactions of mouse brain proteins and thimet oligopeptidase with 14-3-3epsilon and calmodulin. Proteomics.

[73] Berti D.A., Russo L.C., Castro L.M., Cruz L., Gozzo F.C., Heimann J.C., Lima F.B., Oliveira A.C., Andreotti S., Prada P.O. (2012). Identification of intracellular peptides in rat adipose tissue: Insights into insulin resistance. Proteomics.

[74] Fricker L.D., Gelman J.S., Castro L.M., Gozzo F.C., Ferro E.S. (2012). Peptidomic analysis of HEK293T cells: Effect of the proteasome inhibitor epoxomicin on intracellular peptides. J. Proteom. Res..

[75] Ribeiro N.M., Toniolo E.F., Castro L.M., Russo L.C., Rioli V., Ferro E.S., Dale C.S. (2013). AGH is a new hemoglobin alpha-chain fragment with antinociceptive activity. Peptides.

[76] de Araujo C.B., Russo L.C., Castro L.M., Forti F.L., do Monte E.R., Rioli V., Gozzo F.C., Colquhoun A., Ferro E.S. (2014). A novel intracellular peptide derived from g1/s cyclin d2 induces cell death. J. Biol. Chem..

[77] Russo L.C., Araujo C.B., Iwai L.K., Ferro E.S., Forti F.L. (2016). A Cyclin D2-derived peptide acts on specific cell cycle phases by activating ERK1/2 to cause the death of breast cancer cells. J. Proteom..

[78] de Araujo C.B., de Lima L.P., Calderano S.G., Damasceno F.S., Silber A.M., Elias M.C. (2019). Pep5, a fragment of cyclin D2, shows antiparasitic effects in different stages of the Trypanosoma cruzi life cycle and blocks parasite infectivity. Antimicrob. Agents Chemother..

[79] Monte-Silva ERC R.C., Russo L.C., Castro L.M., Gozzo F.C., de Araujo C.B., Peron J.P.S., Rioli V., Ferro E.S. (2016). EL28 is a novel intracellular peptide that activates immune proteasome and CD8+ T-cell response. J. Proteom..

[80] Gelman J.S., Sironi J., Berezniuk I., Dasgupta S., Castro L.M., Gozzo F.C., Ferro E.S., Fricker L.D. (2013). Alterations of the intracellular peptidome in response to the proteasome inhibitor bortezomib. PLoS ONE.

[81] Dasgupta S., Fishman M.A., Mahallati H., Castro L.M., Tashima A.K., Ferro E.S., Fricker L.D. (2015). Reduced Levels of Proteasome Products in a Mouse Striatal Cell Model of Huntington’s Disease. PLoS ONE.

[82] Morozov A.V., Karpov V.L. (2018). Biological consequences of structural and functional proteasome diversity. Heliyon.

[83] Russo L.C., Castro L.M., Gozzo F.C., Ferro E.S. (2012). Inhibition of thimet oligopeptidase by siRNA alters specific intracellular peptides and potentiates isoproterenol signal transduction. FEBS Lett.

[84] Cavalcanti D.M., Castro L.M., Rosa Neto J.C., Seelaender M., Neves R.X., Oliveira V., Forti F.L., Iwai L.K., Gozzo F.C., Todiras M. (2014). Neurolysin knockout mice generation and initial phenotype characterization. J. Biol. Chem..

[85] Castro L.M., Cavalcanti D.M., Araujo C.B., Rioli V., Icimoto M.Y., Gozzo F.C., Juliano M., Juliano L., Oliveira V., Ferro E.S. (2014). Peptidomic analysis of the neurolysin-knockout mouse brain. J. Proteom..

[86] Ramachandran K.V., Margolis S.S. (2017). A mammalian nervous-system-specific plasma membrane proteasome complex that modulates neuronal function. Nat. Struct. Mol. Biol..

[87] Mechoulam R., Hanus L.O., Pertwee R., Howlett A.C. (2014). Early phytocannabinoid chemistry to endocannabinoids and beyond. Nat. Rev. Neurosci..

[88] Wang P., Zheng T., Zhang M., Xu B., Zhang R., Zhang T., Zhao W., Shi X., Zhang Q., Fang Q. (2018). Antinociceptive effects of the endogenous cannabinoid peptide agonist VD-hemopressin(beta) in mice. Brain Res. Bull..

[89] Zheng T., Zhang R., Zhang T., Zhang M.N., Xu B., Song J.J., Li N., Tang H.H., Wang P., Wang R. (2017). CB1 cannabinoid receptor agonist mouse VD-hemopressin(alpha) produced supraspinal analgesic activity in the preclinical models of pain. Brain Res..

[90] Recinella L., Chiavaroli A., Ferrante C., Mollica A., Macedonio G., Stefanucci A., Dimmito M.P., Dvoracsko S., Tomboly C., Brunetti L. (2018). Effects of central RVD-hemopressin(alpha) administration on anxiety, feeding behavior and hypothalamic neuromodulators in the rat. Pharmacol. Rep..

[91] Leone S., Recinella L., Chiavaroli A., Martinotti S., Ferrante C., Mollica A., Macedonio G., Stefanucci A., Dvoracsko S., Tomboly C. (2017). Emotional disorders induced by Hemopressin and RVD-hemopressin(alpha) administration in rats. Pharmacol. Rep..

[92] Mechoulam R. (1986). Interview with Prof. Raphael Mechoulam, codiscoverer of THC. Einstein. Int. J. Addict..

[93] Mechoulam R. (2010). [Endocannabinoids and psychiatric disorders: The road ahead]. Braz J. Psychiatr..

[94] Mechoulam R., Shani A., Edery H., Grunfeld Y. (1970). Chemical basis of hashish activity. Science.

[95] Blair R.E., Deshpande L.S., Sombati S., Falenski K.W., Martin B.R., DeLorenzo R.J. (2006). Activation of the cannabinoid type-1 receptor mediates the anticonvulsant properties of cannabinoids in the hippocampal neuronal culture models of acquired epilepsy and status epilepticus. J. Pharmacol. Exp. Ther..

[96] Jones N.A., Hill A.J., Smith I., Bevan S.A., Williams C.M., Whalley B.J., Stephens G.J. (2010). Cannabidiol displays antiepileptiform and antiseizure properties *in vitro* and *in vivo*. J. Pharmacol. Exp. Ther..

[97] Shafaroodi H., Samini M., Moezi L., Homayoun H., Sadeghipour H., Tavakoli S., Hajrasouliha A.R., Dehpour A.R. (2004). The interaction of cannabinoids and opioids on pentylenetetrazole-induced seizure threshold in mice. Neuropharmacology.

[98] Wallace M.J., Martin B.R., DeLorenzo R.J. (2002). Evidence for a physiological role of endocannabinoids in the modulation of seizure threshold and severity. Eur. J. Pharmacol..

[99] Hua T., Vemuri K., Pu M., Qu L., Han G.W., Wu Y., Zhao S., Shui W., Li S., Korde A. (2016). Crystal Structure of the Human Cannabinoid Receptor CB1. Cell.

[100] Hildebrandt A.K., Dietzen M., Lengauer T., Lenhof H.P., Althaus E., Hildebrandt A. (2014). Efficient computation of root mean square deviations under rigid transformations. J. Comput. Chem..

[101] Maiorov V.N., Crippen G.M. (1994). Significance of root-mean-square deviation in comparing three-dimensional structures of globular proteins. J. Mol. Biol..

[102] Turski W.A., Cavalheiro E.A., Schwarz M., Czuczwar S.J., Kleinrok Z., Turski L. (1983). Limbic seizures produced by pilocarpine in rats: Behavioural, electroencephalographic and neuropathological study. Behav. Brain Res..

[103] Rioli V., Kato A., Portaro F.C., Cury G.K., te Kaat K., Vincent B., Checler F., Camargo A.C., Glucksman M.J., Roberts J.L. (1998). Neuropeptide specificity and inhibition of recombinant isoforms of the endopeptidase 3.4.24.16 family: Comparison with the related recombinant endopeptidase 3.4.24.15. Biochem Biophys. Res. Commun..

[104] Heimann A.S., Gupta A., Gomes I., Rayees R., Schlessinger A., Ferro E.S., Unterwald E.M., Devi L.A. (2018). Generation of G protein-coupled receptor antibodies differentially sensitive to conformational states. PLoS ONE.

[105] Gupta A., Decaillot F.M., Gomes I., Tkalych O., Heimann A.S., Ferro E.S., Devi L.A. (2007). Conformation state-sensitive antibodies to G-protein-coupled receptors. J. Biol. Chem..

[106] Reckziegel P., Festuccia W.T., Britto L.R.G., Jang K.L.L., Romão C.M., Heimann J.C., Fogaça M.V., Rodrigues N.S., Silva N.R., Guimarães F.S. (2017). A novel peptide that improves metabolic parameters without adverse central nervous system effects. Sci. Rep..

[107] Hershko A., Ciechanover A. (1998). The ubiquitin system. Annu. Rev. Biochem..

[108] Groll M., Ditzel L., Lowe J., Stock D., Bochtler M., Bartunik H.D., Huber R. (1997). Structure of 20S proteasome from yeast at 2.4 A resolution. Nature.

[109] Kravtsova-Ivantsiv Y., Ciechanover A. (2012). Non-canonical ubiquitin-based signals for proteasomal degradation. J. Cell Sci..

[110] Goldberg A.L. (2003). Protein degradation and protection against misfolded or damaged proteins. Nature.

[111] Reits E., Griekspoor A., Neijssen J., Groothuis T., Jalink K., van Veelen P., Janssen H., Calafat J., Drijfhout J.W., Neefjes J. (2003). Peptide diffusion, protection, and degradation in nuclear and cytoplasmic compartments before antigen presentation by MHC class I. Immunity.

[112] Kloetzel P.M. (2001). Antigen processing by the proteasome. Nat. Rev. Mol. Cell. Biol..

[113] Kohler A., Bajorek M., Groll M., Moroder L., Rubin D.M., Huber R., Glickman M.H., Finley D. (2001). The substrate translocation channel of the proteasome. Biochimie.

[114] Tian G., Park S., Lee M.J., Huck B., McAllister F., Hill C.P., Gygi S.P., Finley D. (2011). An asymmetric interface between the regulatory and core particles of the proteasome. Nat. Struct. Mol. Biol..

[115] Stumpf M.P., Thorne T., de Silva E., Stewart R., An H.J., Lappe M., Wiuf C. (2008). Estimating the size of the human interactome. Proc. Natl. Acad. Sci. USA.

[116] Sanders A.R., Goring H.H., Duan J., Drigalenko E.I., Moy W., Freda J., He D., Shi J., Gejman P.V. (2013). Transcriptome study of differential expression in schizophrenia. Hum. Mol. Genet..

[117] Fierz B., Chatterjee C., McGinty R.K., Bar-Dagan M., Raleigh D.P., Muir T.W. (2011). Histone H2B ubiquitylation disrupts local and higher-order chromatin compaction. Nat. Chem. Biol..

[118] Norbury C., Nurse P. (1992). Animal cell cycles and their control. Annu. Rev. Biochem..

[119] Malumbres M., Barbacid M. (2005). Mammalian cyclin-dependent kinases. Trends Biochem Sci.

[120] Morgan D.O. (1997). Cyclin-dependent kinases: Engines, clocks, and microprocessors. Annu. Rev. Cell Dev. Biol..

[121] Ohtsubo M., Theodoras A.M., Schumacher J., Roberts J.M., Pagano M. (1995). Human cyclin E, a nuclear protein essential for the G1-to-S phase transition. Mol. Cell. Biol..

[122] Hunter T., Pines J. (1994). Cyclins and cancer. II: Cyclin D and CDK inhibitors come of age. Cell.

[123] Sherr C.J. (1993). Mammalian G1 cyclins. Cell.

[124] Waclaw R.R., Chatot C.L. (2004). Patterns of expression of cyclins A, B1, D, E and cdk 2 in preimplantation mouse embryos. Zygote.

[125] Qie S., Diehl J.A. (2016). Cyclin D1, cancer progression, and opportunities in cancer treatment. J. Mol. Med. (Berl).

[126] Lecker S.H., Goldberg A.L., Mitch W.E. (2006). Protein degradation by the ubiquitin-proteasome pathway in normal and disease states. J. Am. Soc. Nephrol..

[127] Vodermaier H.C. (2004). APC/C and SCF: Controlling each other and the cell cycle. Curr. Biol..

[128] Harper J.W., Burton J.L., Solomon M.J. (2002). The anaphase-promoting complex: it’s not just for mitosis any more. Genes Dev..

[129] Alao J.P. (2007). The regulation of cyclin D1 degradation: Roles in cancer development and the potential for therapeutic invention. Mol. Cancer.

[130] Pagano M., Theodoras A.M., Tam S.W., Draetta G.F. (1994). Cyclin D1-mediated inhibition of repair and replicative DNA synthesis in human fibroblasts. Genes Dev..

[131] Sherr C.J., Roberts J.M. (1999). CDK inhibitors: Positive and negative regulators of G1-phase progression. Genes Dev..

[132] Degterev A., Huang Z., Boyce M., Li Y., Jagtap P., Mizushima N., Cuny G.D., Mitchison T.J., Moskowitz M.A., Yuan J. (2005). Chemical inhibitor of nonapoptotic cell death with therapeutic potential for ischemic brain injury. Nat. Chem. Biol..

[133] Caserta T.M., Smith A.N., Gultice A.D., Reedy M.A., Brown T.L. (2003). Q-VD-OPh, a broad spectrum caspase inhibitor with potent antiapoptotic properties. Apoptosis.

[134] Sodeoka M., Dodo K. (2010). Development of selective inhibitors of necrosis. Chem. Rec..

[135] Fernandez-Salas E., Suh K.S., Speransky V.V., Bowers W.L., Levy J.M., Adams T., Pathak K.R., Edwards L.E., Hayes D.D., Cheng C. (2002). mtCLIC/CLIC4, an organellular chloride channel protein, is increased by DNA damage and participates in the apoptotic response to p53. Mol. Cell. Biol..

[136] Valenzuela S.M., Mazzanti M., Tonini R., Qiu M.R., Warton K., Musgrove E.A., Campbell T.J., Breit S.N. (2000). The nuclear chloride ion channel NCC27 is involved in regulation of the cell cycle. J. Physiol..

[137] Argenzio E., Moolenaar W.H. (2016). Emerging biological roles of Cl- intracellular channel proteins. J. Cell Sci..

[138] Wang P., Zeng Y., Liu T., Zhang C., Yu P.W., Hao Y.X., Luo H.X., Liu G. (2014). Chloride intracellular channel 1 regulates colon cancer cell migration and invasion through ROS/ERK pathway. World J. Gastroenterol..

[139] Tian Y., Guan Y., Jia Y., Meng Q., Yang J. (2014). Chloride intracellular channel 1 regulates prostate cancer cell proliferation and migration through the MAPK/ERK pathway. Cancer Biother. Radiopharm..

[140] Manso J.A., Garcia Rubio I., Gomez-Hernandez M., Ortega E., Buey R.M., Carballido A.M., Carabias A., Alonso-Garcia N., de Pereda J.M. (2016). Purification and Structural Analysis of Plectin and BPAG1e. Methods Enzymol..

[141] Kazerounian S., Uitto J., Aho S. (2002). Unique role for the periplakin tail in intermediate filament association: Specific binding to keratin 8 and vimentin. Exp. Dermatol..

[142] Bausch D., Mino-Kenudson M., Fernandez-Del Castillo C., Warshaw A.L., Kelly K.A., Thayer S.P. (2009). Plectin-1 is a biomarker of malignant pancreatic intraductal papillary mucinous neoplasms. J. Gastrointest. Surg..

[143] Bausch D., Thomas S., Mino-Kenudson M., Fernandez-del C.C., Bauer T.W., Williams M., Warshaw A.L., Thayer S.P., Kelly K.A. (2011). Plectin-1 as a novel biomarker for pancreatic cancer. Clin. Cancer Res..

[144] Katada K., Tomonaga T., Satoh M., Matsushita K., Tonoike Y., Kodera Y., Hanazawa T., Nomura F., Okamoto Y. (2012). Plectin promotes migration and invasion of cancer cells and is a novel prognostic marker for head and neck squamous cell carcinoma. J. Proteom..

[145] Pawar H., Kashyap M.K., Sahasrabuddhe N.A., Renuse S., Harsha H.C., Kumar P., Sharma J., Kandasamy K., Marimuthu A., Nair B. (2011). Quantitative tissue proteomics of esophageal squamous cell carcinoma for novel biomarker discovery. Cancer Biol. Ther..

[146] Vallelian F., Deuel J.W., Opitz L., Schaer C.A., Puglia M., Lonn M., Engelsberger W., Schauer S., Karnaukhova E., Spahn D.R. (2015). Proteasome inhibition and oxidative reactions disrupt cellular homeostasis during heme stress. Cell Death Differ..

[147] Fortes G.B., Alves L.S., de Oliveira R., Dutra F.F., Rodrigues D., Fernandez P.L., Souto-Padron T., De Rosa M.J., Kelliher M., Golenbock D. (2012). Heme induces programmed necrosis on macrophages through autocrine TNF and ROS production. Blood.

[148] Baar M.P., Brandt R.M.C., Putavet D.A., Klein J.D.D., Derks K.W.J., Bourgeois B.R.M., Stryeck S., Rijksen Y., van Willigenburg H., Feijtel D.A. (2017). Targeted Apoptosis of Senescent Cells Restores Tissue Homeostasis in Response to Chemotoxicity and Aging. Cell.

[149] Ferreira J.C.B., Campos J.C., Qvit N., Qi X., Bozi L.H.M., Bechara L.R.G., Lima V.M., Queliconi B.B., Disatnik M.H., Dourado P.M.M. (2019). A selective inhibitor of mitofusin 1-betaIIPKC association improves heart failure outcome in rats. Nat. Commun..

